# Management of refractory disease and persistent symptoms in inflammatory arthritis: qualitative framework analysis of interviews with patients and healthcare professionals

**DOI:** 10.1093/rap/rkae076

**Published:** 2024-06-10

**Authors:** Hema Chaplin, Carol Simpson, Kate Wilkins, Jessica Meehan, Nora Ng, James Galloway, Ian C Scott, Debajit Sen, Rachel Tattersall, Rona Moss-Morris, Heidi Lempp, Sam Norton

**Affiliations:** Health Psychology Section, Institute of Psychiatry, Psychology and Neuroscience, King’s College London, London, UK; Centre for Rheumatic Diseases, Department of Inflammation Biology, King’s College London, London, UK; Centre for Rheumatic Diseases, Department of Inflammation Biology, King’s College London, London, UK; Health Psychology Section, Institute of Psychiatry, Psychology and Neuroscience, King’s College London, London, UK; Rheumatology Department, Guy’s and St Thomas’ NHS Foundation Trust, London, UK; Centre for Rheumatic Diseases, Department of Inflammation Biology, King’s College London, London, UK; Rheumatology Department, King’s College Hospital NHS Foundation Trust, London, UK; Primary Care Centre Versus Arthritis, School of Medicine, Keele University, Keele, UK; Haywood Academic Rheumatology Centre, Haywood Hospital, Midlands Partnership University NHS Foundation Trust, Stoke-on-Trent, UK; Rheumatology Department, University College London Hospitals NHS Foundation Trust, London, UK; Versus Arthritis Centre for Adolescent Rheumatology, University College London, London, UK; Rheumatology Department, Sheffield Teaching Hospitals NHS Foundation Trust, Sheffield, UK; Barbara Ansell National Network for Adolescent and Young Adult Rheumatology, UK; Health Psychology Section, Institute of Psychiatry, Psychology and Neuroscience, King’s College London, London, UK; Centre for Rheumatic Diseases, Department of Inflammation Biology, King’s College London, London, UK; Health Psychology Section, Institute of Psychiatry, Psychology and Neuroscience, King’s College London, London, UK; Centre for Rheumatic Diseases, Department of Inflammation Biology, King’s College London, London, UK

**Keywords:** multidisciplinary team, persistent symptoms, polyarticular JIA, refractory, rheumatoid arthritis, qualitative

## Abstract

**Objectives:**

This study aims to explore patients’ and clinicians’ experiences in managing and living with refractory disease (RD) and persistent physical and emotional symptoms (PPES) in patients with RA or polyarticular JIA from their perspectives through interviews and/or focus groups.

**Methods:**

A qualitative exploration with 25 patients and 32 multidisciplinary rheumatology healthcare professionals (HCPs) was conducted to obtain participants respective understanding and experiences of managing RD/PPES and its impact on the patient–professional relationship. A pragmatic epistemology approach with framework analysis was employed.

**Results:**

Four key themes were identified from both patients and professionals in the management of RD/PPES: risk/perpetuating factors/triggers; need for a patient-centred holistic approach to care, diagnosis and treatment; discordance and impact on the patient–practitioner relationship and current problems in managing RD/PPES. These themes covered 22 subthemes, with none being patient specific and seven being HCP specific. Suggestions for potential management strategies were highlighted throughout, such as involving other specialties or a multidisciplinary team, assessing/treating patient-reported outcome measures and psychosocial factors, patient (re)education, need for adjustments/aids or adaptations, checking the diagnosis and further investigations/imaging and optimizing medications.

**Conclusion:**

Management strategies need to be developed that enable appropriate treatment plans for those with RD/PPES that account for wider biopsychosocial factors beyond inflammation and reduce discordance in the patient–practitioner relationship.

Key messages

- This is the first qualitative study with patients and clinicians to explore refractory disease and persistent symptoms management.
- A holistic approach to diagnosis/treatment is important, with an impact on the patient–healthcare professional relationship occurring if management expectations are misaligned.
- Potential management strategies are highlighted, e.g. multidisciplinary team involvement, assessing/treating patient-reported outcome measures, patient (re)education and optimizing medications.

## Introduction

Persistent RA or polyarticular JIA (pJIA) and its treatment can impact a person’s quality of life. The best treatment following multiple inadequate responses to DMARDs is uncertain [1, 2], such as for those with refractory disease (RD). Even in those who achieve good response, reductions in persistent emotional and physical symptomology (PPES) such as pain and fatigue remain to be achieved [3, 4]. A greater knowledge and understanding of the person who lives with persistent RA/pJIA in relation to their inflammation (RD) or symptoms (PPES) is important to improve management [5]. Therefore, qualitative exploration to identify any potential management strategies that have been employed by multidisciplinary healthcare professionals (HCPs) and patients is required. Understanding wider HCP perspectives in the management of patients with RD may explain some of the current clinical limitations and how these can be addressed [6].

There are discordances between patients and HCPs about clinical outcomes and priorities [7, 8], with patients concerned with psychosocial effects and HCPs favouring physical effects. Therefore, it is important to determine whether patients’ and HCPs’ views converge or diverge. Patients report that they are not listened to and lose confidence both in themselves and their physicians when experiencing disparities in patient–physician priorities in consultations, with patients desiring more support for their quality of life while rheumatologists are more inflammation focused [8, 9]. Therefore, the impact on the patient–HCP relationship and any discordances in outcomes/priorities will be examined. Given this gap in the literature for managing the complex nature of RD/PPES across RA and pJIA, this qualitative study aimed to understand the experiences of managing RD/PPES from both the patients’ and HCPs’ perspectives, including determining treatment expectations and experiences and the impact on the patient–HCP relationship.

## Methods

### Design and sample

A qualitative study was conducted with one-on-one patient interviews and interviews and focus groups with multidisciplinary rheumatology HCPs, following a pragmatic epistemology approach [10, 11]. Both patients and HCPs were recruited from three London and one Midlands rheumatology outpatient clinics, with additional HCPs recruited from a national UK network of rheumatology HCPs (see Supplementary Table S1, available at *Rheumatology Advances in Practice* online, for criteria). To maintain anonymity, recruitment sites are reported as A–E and aggregate data are presented [12]. Full National Health Service ethical approval was granted by the London–Hampstead Research Ethics Committee (18/LO/1171, June 2018). Braun and Clarke [13] suggest 20–30 interviews for a medium–large PhD research project, due to the resources and skills involved. Patients were purposively recruited [14], stratified during screening to identify those with PPES [low 28-joint DAS (DAS28)/10-joint juvenile arthritis disease activity score (JADAS10) but high physician’s global assessment (PGA)] or RD (high DAS28/JADAS10) based on their clinical notes [15–18] to ensure appropriate representation. When patients were approached, they were asked whether they had persistent symptoms lasting ≥3 months affecting functioning [19], confirming eligibility.

Rather than sampling until data saturation, an information power approach was followed [20]. In this study, the aim is broad and the interview dialogue may be weaker, as performed by a junior researcher (which reduces information power), however, the participants are highly specific for the study aim, supported partially by cognitive-behavioural theory, and comparative analysis was conducted (which increases information power), thus in line with a medium-sized sample [13], and information gained was continuously appraised.

### Data collection and analysis

The audio recorded data were gathered by H.C. through face-to-face or telephone interviews/focus groups at one time point, using a semi-structured interview guide [21] (see Supplementary Data S1, available at *Rheumatology Advances in Practice* online). Transcripts were not returned due to a lack of time/financial resources, with other validation/credibility checks conducted [22]. H.C. had relevant qualitative training and research experience. Both patients and HCPs completed a sociodemographic questionnaire, with the Musculoskeletal Health Questionnaire (MSK-HQ [23]) completed by patients and descriptive statistics reported.

Reporting follows the Consolidated Criteria for Reporting Qualitative Research (COREQ) guidance [24] (see Supplementary Table S2, available at *Rheumatology Advances in Practice* online) to ensure transparency. Three coders (H.C., J.M. and H.L.) discussed initial codes and themes to ensure consistency and credibility across interpretations. Framework analysis was chosen as a comprehensive review of collected narratives, driven by participants’ original accounts, and provided an in-depth systematic analysis between and within patient and multidisciplinary HCP data [25, 26]. In framework analysis, data are sifted, charted and sorted into key themes using five steps [11, 27]: familiarization, preliminary thematic analysis, application of themes to the dataset systematically, reducing data into summaries and organizing these in a matrix (participants by themes) and identifying patterns/relationships. Interview transcripts were imported into NVivo 12 (Lumivero, Denver, CO, USA) for coding during the initial thematic analysis. The coding frame was then applied to interview transcripts in Word and transferred to an Excel spreadsheet (both Microsoft, Redmond, WA, USA) to enable greater transparency and comparison [28].

### Patient involvement

Patient involvement was integral to the study design and conduct, which evolved with numerous people within adolescent and adult rheumatology and revealed that RD and PPES are problems across both conditions. Therefore, it was decided that patients with pJIA ≥16 years of age and paediatric/adolescent HCPs should be included to explore the understanding of refractory pJIA in addition to adults with RA. The study’s patient research partners (C.S. and K.W.) were consulted to ensure patient perspectives were represented throughout [29], including approving and reviewing patient documents and the interview schedule.

## Results

Of the 60 eligible patients approached, 26 consented and 25 completed interviews. A total of 20 people living with RA (80%) (including one partner) and 5 adults with pJIA (20%) who had RD (*n* = 21) or PPES (*n* = 4) were interviewed. Aggregate sociodemographic and clinical patient characteristics are reported in Table 1. A total of 59 HCPs were contacted; 33 consented and 32 participated (see Supplementary Table S3, available at *Rheumatology Advances in Practice* online). Five focus groups (*n* = 20) and 12 individual interviews were conducted from 11 Hospital Trusts, with 7 outside of London. Professionals represented rheumatology (consultant and registrar), clinical specialist nursing, psychology, physiotherapy, occupational therapy, podiatry, pharmacy and social work. The mean years of rheumatology experience was 11.72 (s.d. 7.14), demonstrating experienced clinicians. The majority received adult (71.9%) or specific musculoskeletal training (84.4%).

**Table 1.** Patient sociodemographic and clinical characteristics

Characteristics	Aggregate averages (*N* = 25)
Sociodemographic	
Age, years, median (IQR)	59 (32)
Female, %	84
Ethnicity, %	
White British	76
White Irish	4
Black/Black British	8
Asian/Asian British	8
Mixed	4
Birthplace UK, %	84
Had to stop/modify education/employment due to RA/JIA, %	64
Registered disabled, %	64
MSK-HQ, mean (s.d.)	
Total score (out of 56)	24.6 (9.89)
Days physically active (out of 7)	1.0 (1.54)
Clinical	
Inflammatory arthritis diagnosis	
RA	20
pJIA	5
Disease duration, years, median (IQR)	20 (14)
Age at diagnosis, years, median (IQR)	28 (29)
RF positive, %	72
Disease activity (DAS28 score), %	
Remission (< 2.6)	0
Low (≥2.6–≤3.2)	16
Moderate (>3.2–≤5.1)	60
High (>5.1)	24
Time to first DMARD, months, median (IQR)	6.75 (14.6)
Previous total DMARDs, *n*, mean (s.d.)	6.24 (2.59)
Previous csDMARDs, *n*, mean (s.d.)	2.92 (1.35)
Previous b/tsDMARDs, *n*, mean (s.d.)	3.32 (1.68)

Time to first DMARD was either from symptom onset or diagnosis, depending on the available data recorded in the medical notes.

### Themes for management of RD/PPES

**Figure 1.**
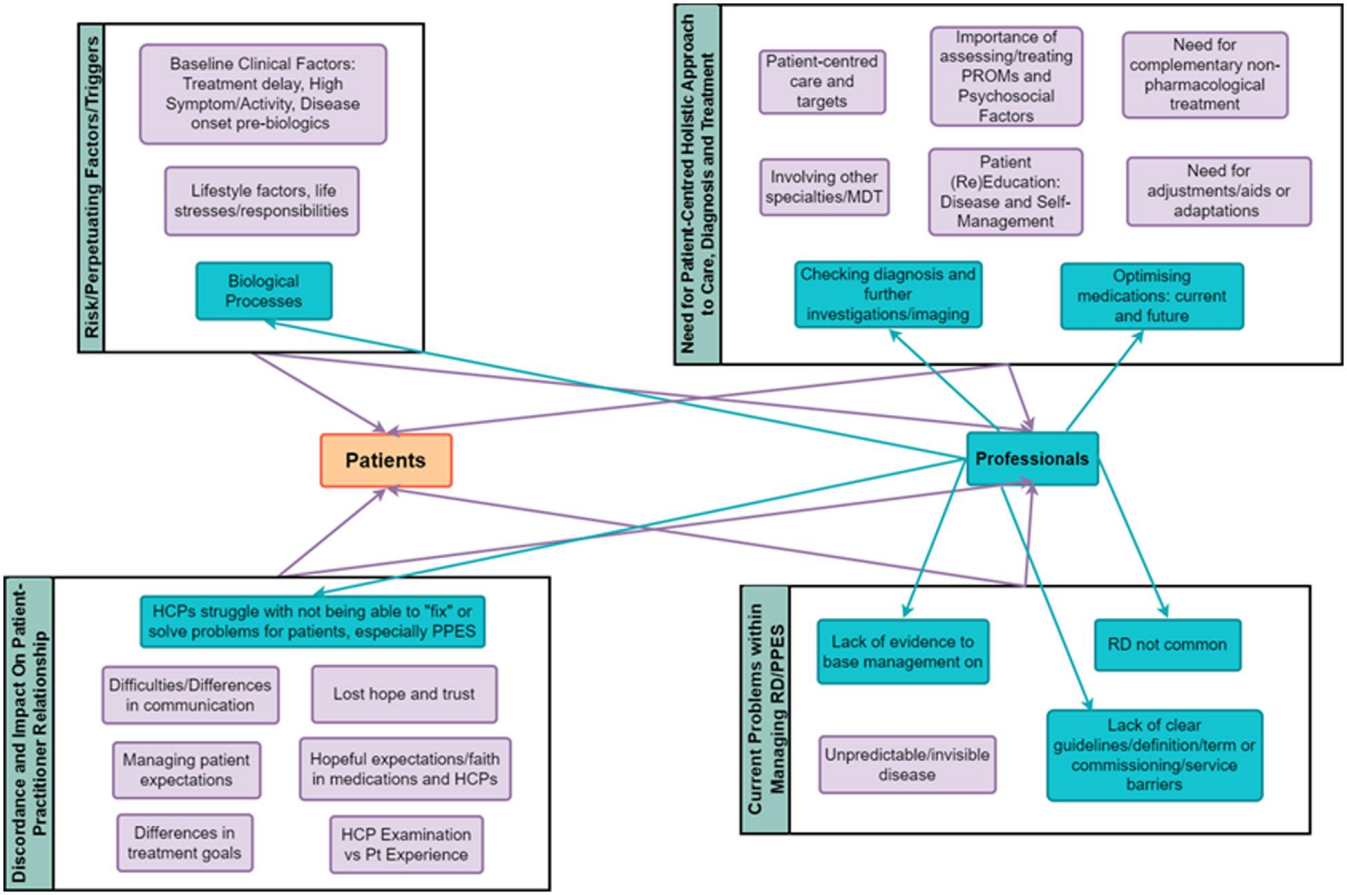
Thematic map of managing RD/PPES identified by patients and HCPs

Experiences and management strategies of RD/PPES are presented in Fig. 1 (see Supplementary Data S2, available at *Rheumatology Advances in Practice* online), highlighting similarities and differences between patients and HCPs. Four key themes were identified from both patients and HCPs regarding the management of RD/PPES: risk/perpetuating factors/triggers; the need for a patient-centred holistic approach to care, diagnosis and treatment; discordance and impact on the patient–practitioner relationship and current problems in managing RD/PPES. These themes covered 22 subthemes, with none being patient specific and 7 being HCP specific. From the framework constructed during analysis, patterns across the data and participants were explored (see Supplementary Fig. S1, available at *Rheumatology Advances in Practice* online).

Those with RA-RD mainly mentioned ‘differences in treatment goals’, compared with the other three patient groups, although generally information from patients was allocated to the ‘difficulties/differences in communication’ and ‘hopeful expectations in medications and HCPs’ subthemes and less attributed to the ‘risk/perpetuating factor/triggers’ theme. HCP perspectives were broadly interpreted and aligned with all the themes compared with patients. There were no clear patterns in the management themes or subthemes. Allied health professionals (AHPs) did not mention elements that fit with the ‘optimizing medications’ or ‘checking diagnosis’ subthemes, as these clinical decisions are not part of their professional role.

### Risk/perpetuating factors/triggers

This theme comprised three subthemes focusing on risks and triggers: baseline clinical factors, lifestyle factors and life stresses/responsibilities and the role of biologic processes (see Table 2). Both patients and HCPs reported clinical factors were important at symptom onset/diagnosis in explaining elements that triggered or worsened RA/pJIA. This included treatment or diagnosis delay, experiencing severe symptoms or disease activity, infection or disease onset pre-biologics. Lifestyle factors and stresses/responsibilities were mentioned by both patients and HCPs as triggers for RA/pJIA flaring/starting and could be understood as precipitating or perpetuating factors.

**Table 2.** Illustrative accounts for theme 1: risk/perpetuating factors/triggers

Subthemes	Patients’ Accounts	HCPs’ Accounts
Baseline clinical factors	‘[Doctors] treated me for carpal tunnel and all sorts of different things. Erm, it took a while to, erm, diagnose the RA, which I don’t know why [diagnosis] takes so long when it’s, you know, it’s a blood test…each stick to their own field so [the doctor] knew something was going on but he didn’t know it was RA’. — PAT1C (female, RA)	‘[Patients with RD] tend to have very severe inflammation from the outset, they tend to have high joint counts…often have evidence or erosive damage when you first meet them’. —HCP1B (male, consultant rheumatologist) ‘I’ve seen patients who’ve been diagnosed years down the line and if they’d perhaps been referred through to OT or physio earlier, [patients] might have coped a little bit better with their condition, or if they been given advice on how to manage differently…they may have been a little bit more functional’. — HCP7C (female, occupational therapist)
Lifestyle factors and life stresses or responsibilities	‘It came after the death of me father when [RA] kicked in, and I mean, I can’t prove it’s that but it, it’s, but it’s happened again after my mother died and she died in 2003 and there seems to be a couple of year’s gap after both of them died and then [inflammation] kicks in (hmm). After my mother died, it just, [RA] just went sky-high and it’s been like that ever since’. — PAT5C (female, RA) ‘There are triggers to RA such as stress and diet and, to some extent, exercise…I’ve got four brothers and sisters, but they’re all [living] somewhere else (laughs) so [caring for father] falls to me, so yeah, [stress/caring] does impact on RA a lot’. — PAT4B (female, RA)	‘Is [RD/PPES] to do with modern life as well? On phones, and tapping on keyboards that 20 years ago not everyone did that all day long, putting more stress through your joints’. — HCP5B (female, pharmacist) ‘There’s a lot of social and family complexities at home, perhaps there's been a marriage breakdown or a death in the family or all things like that and they’re not coming to appointments. Those for me would be a worry to think well, actually maybe we [HCPs] need to safeguard this young person, erm, before their disease becomes more active and we can’t control it’. — HCP3D (female, social worker)
Role of biological processes		‘There’s some people who, for whatever reasons, whether that’s genetic, whether it’s different cytokines driving their disease, age, gender, other conditions, have truly refractory disease, erm, and I think that's likely to be predominantly genetic factors and disease-specific factors, erm, you know, seropositivity, some of those things’. — HCP4C (female, consultant rheumatologist) ‘There are underlying physiological, maybe neuroscientific reasons why some people are more vulnerable to chronic, unexplained symptoms than others. I don’t have any sense that [PPES is] purely psychological, erm, I think that certain people’s bodies and brains are just more vulnerable to chronic symptoms than others’. — HCP1A (female, psychologist)

Stress was commonly reported, covering cognitive/psychological, financial and physical stresses, with patients also mentioning diet, obesity and pregnancy as contributing factors. HCPs remarked that patients often had other caregiving responsibilities, meaning they were unable to focus on their own healthcare. Paediatric HCPs also implied the family environment was important for young people as another trigger of stress or support. A minority of HCPs mentioned biologic processes such as genetics or physiological vulnerability as predisposing factors for developing more severe RA/pJIA or PPES, particularly pain processing mechanisms.

### Need for a patient-centred holistic approach to care, diagnosis and treatment

The most common theme was the need for a holistic care approach covering eight subthemes: patient-centred care and targets; involving other specialties/multidisciplinary team (MDT); importance of assessing/treating patient-reported outcome measures (PROMs) and psychosocial factors; need for complementary non-pharmacological treatment; patient (re)education: disease and self-management; need for adjustments/aids or adaptations, with two HCP-specific subthemes: checking diagnosis and further investigations/imaging; and optimizing medications: current and future (see Table 3). One cross-cutting subtheme was to identify targets important to patients, as they are experts in living with their disease.

**Table 3.** Illustrative accounts for theme 2: need for a patient-centred holistic approach to care, diagnosis and treatment

Subthemes	Patients’ accounts	HCPs’ accounts
Patient-centred care and targets	‘You can talk to the consultants and tell them but they don’t know what is going on in your body (hmm). They can only tell by blood tests (yep) if [RA’s] active, so they do a blood test and [rheumatologist] goes: “Oh yeah, your white blood cells are up, [RA] is a bit active.” But you as a person who’s got the disease know it’s active’. — PAT4D (female, RA) ‘Obviously if [patient and rheumatologist] are talking about swapping medication or anything like that err then yeah, no absolutely, they [HCP] value, err or we talk about what my err opinion would be of that [medication], you know would I be ok to try this out? Yeah it’s a discussion rather than them talking and me listening type thing’. — PAT2D (male, pJIA)	‘What exactly is the target? Often with treat-to-target you don’t often include things like the patient’s quality of life, additional symptoms, function. It’s just how many swollen joints, how many tender joints, what’s their ESR and how are [patients] generally feeling and you can miss out on a lot of things…if you had more time and you could perhaps pick through some of the details of what really matters to that patient, and that patient’s ideas about that then, you know, you could get a better [clinical] outcome’. — HCP6D (male, rheumatology registrar) ‘Really try to understand it from the patient’s perspective, where they are in their understanding and try to meet them’. — HCP1A (female, psychologist)
Involving other specialties or MDT	‘People with RA have other physical problems as well and some stemming as a result of the RA and it’s simply not helpful to have them off into separate specialisms and then to refuse to think about them’. — PAT5D (female, RA) ‘Help you with your, the mental side of [RA] more so than the [physical]. They do a brilliant job on the physical side, but they need to get the ball rolling with somebody, even if they put you forward, see somebody, you know, about, the emotional and mental side of [RA]’. — PAT4C (male, RA)	‘So if it’s just hand pain that the person’s complaining of and we [HCPs] feel we’ve had good results erm or we’re struggling to get efficacy, I can refer to OT to say hand therapy. Physio, we’ve unfortunately we [HCPs] have got quite a wait’. — HCP6C (female, clinical nurse specialist) ‘We can’t actually as doctors do much more unless the other parts of the package are accepted as well (agreement), so that’s the psychology, the OT, the physio, all those parts of the package also need to be accepted…there won’t be one magic bullet for this’. — HCP4E (female, consultant rheumatologist)
Importance of assessing or treating PROMs and psychosocial factors	‘The questionnaire will ask you, what, how are you feeling emotionally or mentally, well what’s the point in asking that if you’re not gonna suggest how to (laughs) to manage it [mental health]. You know if you are feeling moderately depressed or down or mildly depressed or down, what information can you offer me to deal with that [depression]?’ — PAT1A (female, RA) ‘I think first off, I think that mentally, that should be spoken about more. I think emotionally, a lot of people think of a chronic illness and will only think of the physical pain’. — PAT6C (female, pJIA)	‘The benefit of the PROMs is that you can kind of, that you can see change a bit more easily, although some of [responses] don’t fluctuate that much but like, it’s quite nice as like a measure that if you see them [changes], cos there’s so many different people seeing the same person’. — HCP3B (male, rheumatology registrar) ‘Understanding the context that the young person lives in as well and having a good psychosocial understanding of that young person, so you’ve got the pain and their disease or condition, that’s one thing. But also often if you find out a little bit more about family life, school life, friend life and sort of sexuality and identity, all those sorts of things [wider psychosocial context], you quite often find there are other stresses that are kind of the push factor for young people’. — HCP3D (female, social worker)
Need for complementary non-pharmacological treatment	‘Self-help like hydrotherapy, which is the best thing [treatment] for this disease to be quite honest than lots more other stuff. That’s what I think anyway because none of it seems to really work. You take painkillers. Erm I mean erm what’s methotrexate? It just lowers your immune system and I know it [methotrexate] goes with all the others, but at the end of the day it’s a bit toxic. They’re all toxic’. — PAT2A (male, RA) ‘Like they know I can’t really exercise, so, maybe erm, trying to do [exercise] in water or different small exercises. Dr [anonymous] has given me ideas of things [exercises] I can do just sitting in the chair’. — PAT3B (female, RA)	‘General mobility exercises, trying to maintain some strength, erm, [I have] had a couple that I’ve put in the pool as well, hydrotherapy, and that will be maintenance, like let’s not get any stiffer. Let’s not get any weaker. Let’s not miss too much school…kind of keep a routine without exhausting themselves too much or overdoing it but still be able to [maintain function]’. — HCP7D (female, physiotherapist) ‘We [podiatrists] give lifestyle, we give pacing, we give footwear, we give advice on perhaps changing jobs, we give advice on if your feet are painful, just the simple stuff, perhaps ice or, erm, using a method whether it be ice or warmth, whichever one suits them best to try and reduce some of the inflammation over those areas…we also give their traditional smoking advice, weight loss advice, the holistic sort of advice’. — HCP1C (female, podiatrist)
Patient (re)education: disease and self-management	‘My mum and dad were just told “oh she’s got arthritis, she’s gonna stay here in the night”. And then after that “it was ok, she can go home, she’s gonna start this medication”, and there was no explanation about the illness or any advice on what [HCPs] could do to help’. — PAT6C (female, pJIA) ‘They’re very good at the hospital at giving out leaflets and information. [HCPs] always have, you know, whether a new drug, what they normally do is say this new is on the market. Take this away. Go and read the leaflets. Go and do your own research and then if you fancy switching let’s come back and talk about [changing medication] and we see how we go about it’. — PAT6D (female, RA)	‘Sometimes challenging to help [patients] to shift the focus from the symptoms and understand that their rheumatoid is not flaring, is not out of control—there’s something else that needs to be addressed in a completely different way and might not respond as easily as the rheumatoid’. — HCP2A (male, clinical nurse specialist) ‘Patients haven’t got an understanding of the condition and perhaps, sort of how to manage [IA/symptoms]…to actually teach [patients] how to live with their condition 24/7…joint education with them, which is showing them different ways of doing [activities] so they’re not putting so many stresses and strains throughout the joints’. — HCP7C (female, occupational therapist)
Need for adjustments, aids or adaptations	‘When I stay at someone else’s house or in a hotel, I can’t open the door, I can’t turn a key, I can’t use the taps, so I’m very conscious that I’m fine in my own home because everything is touch sensitive and I don’t have [items] that require sort of manual dexterity’. — PAT1B (female, RA) ‘[Partner]: they have given you a little aid, a finger, little support has been issued from time to time, you know, like help you, they [HCPs] come up with ideas how to open a milk bottle’ [Partner]. — PAT5A (female, RA) ‘My dad is disabled, he used to use some different equipment in the bathroom so that was useful for me as well when I had my joints [flaring] in the beginning. So, he had like for the toilet, he had this thing [toilet frame], it’s a handle on both sides so I used to use it, that was useful’. — PAT9D (female, pJIA)	‘Sometimes we [HCPs] can show people assistive equipment and sometimes we can order that or people can go and some of the small sort of kitchen type equipment like bottle openers, jar openers they can go away and purchase those privately’. — HCP8C (female, occupational therapist) ‘[Patients] can do like hand wax [treatment] and splints. They can then have equipment demonstrated to help with activities of daily living. The patients can adapt to how their hands have gone [changed]’. — HCP6C (female, clinical nurse specialist) ‘The visibility of it [RA/aids], um, you know, choices about whether to use, use aids to walk, that kind of thing. Um, and, you know often there’s quite a lot of discourse about being defined by the RA’. — HCP6B (female, psychologist)

Subthemes	HCPs’ accounts	
Checking diagnosis and further investigations or imaging	‘Sometimes those patients have quite a subtle arthritis and that it’s difficult to pick up clinically, so it’s not often very swollen joints or even on ultrasound scan they might be not obvious on ultrasound scan but then get picked up on more invasive or detailed testing like MRI scans then…[patients] probably get imaged more regularly when you try and separate out is it the treatment that’s working or is it something else? Is it inflammation? Is there something else that is causing the refractory pain? I would say, I would image them more often, especially if they’ve been through a few medications and you want to know am I going to change on to yet another medication’. — HCP4E (female, consultant rheumatologist) ‘Particularly talking sort of neck, TMJ, hip, you know, where you can’t see swelling, you probably image them a lot more in those, where you wouldn’t, you know, with a poly flare you wouldn’t do [additional imaging]’. — HCP3E (female, consultant rheumatologist) ‘I guess another thing that I should touch on it is have we [rheumatologists] got the diagnosis right? Erm, you know, you don’t want to, cos there are other causes of inflammatory arthritis, such as crystal arthritis (hmm) …have you got the correct diagnosis, erm, is another important consideration, always to think back if someone’s truly not responding [to treatment]’. — HCP3C (male, consultant rheumatologist)	
Optimizing medications: current and future	‘One thing which does interest me is the fact that there is so much reliance on biologics particularly from some of my younger consultant colleagues that people ignore the old-fashioned disease-modifying agents and they’re licensed for a reason, it’s cos they work. Erm, but the number of people that have been on eight different biologics, but nobody’s actually thought to try gold or penicillamine, erm, and it’s quite interesting that people, you know, because we [rheumatologists] don’t use them routinely, then people don’t use them [csDMARDs] and, you know, not often but on occasion, you know, any response to a DMARD or a biologic is an individual response and I think there are patients that do have options, which are simply ignored because people aren’t used to those drugs anymore’. — HCP2C (male, consultant rheumatologist) ‘[Selecting appropriate treatments] gonna be more difficult because we’ve got more different drugs and whilst that sounds counterintuitive, doesn’t it, cos the more you’ve got in your armoury, the better [treatment options] should be, but actually I think it’ll make it worse’. — HCP6E (female, clinical nurse specialist) ‘Sometimes we [rheumatologists] switch it [medication] to intravenous therapy and see whether that works and some children it actually, we’ve given [medication in a different administration] and sometimes actually we’ve got people to come for supervised injections for a good few months (HCP8E: yeah, absolutely) but actually for that particular kid it [changing medication administration] didn’t make any difference’. — HCP9E (female, consultant rheumatologist)	

HCPs reflected that the treat-to-target approach misses important targets for patients in determining treatment response. Involving other specialties and having an MDT was key to supporting patients comprehensively, including nursing, physiotherapy, hand therapy, occupational therapy, pain clinic/service and orthopaedic surgery, depending on their needs. Both recognized that rheumatologists were important for managing medications and physical elements, while other specialties were relevant in providing specialist care for functional, emotional and psychological aspects. Another highlighted problem was that different departments often work in ‘silos’, which negatively affects patients with multimorbidity.

The importance of assessing and treating PROMs and psychosocial factors was discussed by both. Standardized measures are useful to monitor progression and changes in patient well-being. However, it was recognized that PROMs alone may not fully capture the complexity of experiences, requiring in-depth discussion. Psychosocial factors, in particular, need to be assessed and managed to acknowledge the impact of psychosocial problems that influence other elements, e.g. pain and disability. Thus, there was a need for non-pharmacological treatment to complement medication, with patients mentioning hydrotherapy as most beneficial. Others included wax/heat therapy, strength/mobility exercises, lifestyle advice, home-based strategies and group-based peer support.

Patients with RD/PPES typically have long-established disease and therefore may require re-education, including new medications and effective coping strategies. This should be done in collaboration with HCPs and patient organizations offering this advice. Especially those who were given information as a child or to their parents, their understanding about their RA/pJIA, symptoms and treatment requires updating when in adulthood. HCPs stated that patients needed education to shift their understanding and focus away from the original inflammatory processes driving disease/symptoms to the role of other influences such as pain sensitization or joint damage that cannot be treated easily with medication and requiring specific self-management techniques and behaviour modifications.

Additionally, the need for adjustments/aids or adaptations to the home or clothing, e.g. opening jars or using insoles, was identified as integral to continue with daily activities such as shopping or cooking. These alterations are helpful to maintain independence and use less energy/effort. Many of the adjustments/aids related to supporting reduced hand function or mobility. However, modifications could be taken too far, where one patient reported they had made so many changes to their home to avoid symptoms that they struggled in other environments. Another factor is the visibility of the recommended aids, with patients resisting their use at first, which feeds into the perception of being defined by their disease.

Rheumatologists stated that they would reconfirm the diagnosis and conduct further investigations/imaging to rule out other conditions and determine subclinical inflammation in joints that are more difficult to assess by physical examination, such as the temporomandibular joints and hips. Optimizing medications included the hope for future stratified/personalized medicine. In the meantime, rheumatologists could better optimize the use of current DMARD options. Optimization can be achieved by monitoring adherence, changing the administration method (e.g. from injection to infusion) or reintroducing previously successful conventional synthetic DMARDs (csDMARDs) (if not intolerant) or historical csDMARDs.

### Discordance and impact on patient–practitioner relationship

The third theme focused on the discordances between patients and practitioners regarding communication, treatment and expectations that led to tensions in the patient–practitioner relationship, incorporating seven subthemes: clinical presentation *vs* patient perception/experience, differences in treatment goals, managing patient expectations, lost hope and trust, difficulties/differences in communication, hopeful expectations/faith in medications and HCPs and HCPs’ struggle with not being able to ‘fix’ or solve problems for patients, especially PPES (see Table 4). The difference in clinical presentation or physician assessment and patients’ perception was a source of disagreement and tension.

**Table 4.** Illustrative accounts for theme 3: discordance and impact on patient–practitioner relationship

Subthemes	Patients’ accounts	HCPs’ accounts
Clinical presentation *vs* patient perception or experience	‘I’ve always found really difficult in that I’ll go into an appointment and [HCPs]’ll say: “Oh, your bloods are fine, so everything is ok” and I’ll be sat there, in a lot of pain and, you know, I won’t feel fine and everyone says it [the news of bloods being fine] with a smile on their face like: “This is great news.” And I just burst into tears every time cos it’s not great news for me, it’s just, you know, you feel like: “Am I making this up? you know, everyone else is telling me that they can’t see anything wrong”’. — PAT11D (female, pJIA) ‘[RA symptoms] was then in every single joint: my jaw, my hands, my knees erm. Again, because there wasn’t very much inflammation, I didn’t feel [HCPs] was really listening to me erm they was just telling me take painkillers…all of the sudden [inflammation] just went mad and [RA] was really starting to show, the inflammation was really swelling. Erm everything was ballooning erm yeah, then trying treatment but [HCPs] kept saying there was no erosion, there was no damage’. — PAT6A (female, RA)	‘[HCPs] do the CHAQ scores and some people who are, we were absolutely convinced [patient] don’t have arthritis. So if you’re talking about looking into the refractory symptoms but again and again they’ve got pain. You’ve done your scanning, you’ve done your clinical examination and everything else, but if you look at your outcome measures, the patient reported outcome measures (HCP8E: they’re terrible) and then your pain score is nine’. —HCP9E (female, consultant rheumatologist) ‘I [ultrasound] scan them on a weekly basis and it’s an interesting experience the disparity between what the patient describes, their belief that they have active disease, what I think when I examine them clinically and what I see after I scan in terms of having active inflammation with doppler signal in their joints and these three perceptions are completely different’. — HCP1D (female, consultant rheumatologist)
Differences in treatment goals	‘[Rheumatologist] tries to talk to me more to take on these different [medications] like. But I mean, I said to him [Rheumatologist], I said but it’s alright, I’m getting old, you don’t realise it’s a worry to me. If I were younger I would try these things’. — PAT3A (Female, RA) ‘[RA’s] not about how your fingers feel and stuff like that [RA’s] about how you, your body feels overall, how that’s affecting you, how that’s affecting you here (taps head to indicate mentally) (so mentally), yeah and how [RA’s] stopping you from living a normal life? What is the biggest thing that is bothering you? What can we do to fix that?’ — PAT1A (female, RA)	‘A disconnect sometimes between patient and clinician expectations. Part of this is a disconnect in how we [HCPs] describe [disease/symptoms] and part of it is a disconnect in what we see as a potential solution…the clinician wants to control the disease activity and the patient wants to have a better quality of life’. — HCP1B (male, consultant rheumatologist) ‘Get a bit of resistance understandably from the patient because they’ve actually hit their target and just because their DAS is still a touch higher than you’d want it [DAS] to be, it can be a tricky to try and negotiate increasing treatment with side effects’. — HCP6D (male, rheumatology registrar)
Managing patient expectations	‘I think when you try something new [medication] you don’t want to overplay it [efficacy] cos all [drugs] won’t work for everybody and I think as long as people know upfront that there’s a chance this might work but don’t be terribly disappointed if it [medication] doesn’t’. — PAT6D (male, RA) ‘I remember my doctor saying to me “we’ll hope for the best, but prepare ourselves for the worst”. And I was thinking are you kidding me? Why can't you fix me? But I understand more about autoimmune stuff and just how different people react to [drugs]’. — PAT10D (female, RA)	‘You try to give hope to patients but I guess you have to be realistic as well, and sometimes it’s helping them realise that [symptoms] might take longer to get better is important so [patients] don’t think: “I’m going to start this medication, I should have felt better the next week”’. — HCP5D (female, rheumatology registrar) ‘I’m still optimistic in tone, I would say, but a bit less [optimistic] because just like you said there definitely is some people that the arthritis I don’t think ever really comes under control’. — HCP7E (female, consultant rheumatologist)
Lost hope and trust	‘I haven’t had a great experience. I don’t feel that I was listened to. Early stages I don’t feel that they believed me, it’s always you look ok and it [RA] wasn’t showing swelling to start off with, but probably cos I’ve had a bad experience, yeah, it’s [previous poor experience] just made me a bit negative on the whole thing…Then it’s too late cos the damage is done [to my joints] and there’s no going back once the damage is done and that’s where I feel maybe I’ve been a little bit let down from erm my consultant, which is why I asked for the second opinion because damage was being done and I wasn’t getting any better, if anything I was feeling worse’. — PAT6A (female, RA) ‘The doctor was quite shocked about my treatment I’ve had from a child that have not helped me one bit…I never got no physio, no hand therapy. And I’ve got nothing offered to me to prevent things [damage] from happening like my fingers being disformed, growing like a swan and that. But he offered all those [drugs] to me so, which was like a relief really. I’ve got help coming, even though I’m 27 now (laughs) and all those years I didn’t really get given it [treatment]. Yeah, no help really…I just feel like I’ve been let down’. — PAT4A (female, pJIA)	‘Yeah I think erm from like my experience of the patients that are coming like obviously we [HCPs] are giving a lot of JAKs now at the end of the line. There is a lot of hope that is kind of lost as well so they don’t, like (imitates grumbling) “ohh none of them have worked before”. So I’ve seen that quite a bit actually’. — HCP5B (female, pharmacist) ‘Or [patients] complain by not attending, don’t they? They complain by disengagement, it’s that silent complainer that’s the worst really to engage with, cos you know that [patients are] suffering at home in some way, shape or form but they just don’t want to face up to it [disease/treatment]’. — HCP6E (female, clinical nurse specialist)
Difficulties or differences in communication	‘So I just gotta like get on with it [my life] and I go to all my appointments and ask them what they [HCPs] want to know, but I don’t really sit there and go into my illness with them in depth, if you know what I mean’. — PAT10D (female, RA) ‘I’m never rushed, you know, so I’m able to express my feelings and how I feel’. — PAT7D (female, RA)	‘Cos obviously [HCPs are] doing tender joint counts, I think people often don’t understand the word ‘tender’ (murmurs of agreement) like “Is this tender when I press?” – “No”. But yeah, “Oh but it [joint] hurts though”. Erm, so they, I think [patients] struggle with that’. — HCP4B (female, rheumatology registrar) ‘I’ve definitely had patients get quite irate with me for labelling something as pain and they’re like: “No it’s not pain, it’s sore.” In my eyes it’s obviously the same thing [symptom] but to them it’s hard to unpick’. — HCP2B (male, consultant rheumatologist)
Hopeful expectations/faith in medications and HCPs	‘Hopefully if this one [biologic] carries on that’s great but if it [biologic] doesn’t, then don’t worry about it, there are other things [drugs] in place. So you get that confidence with that as well…you build your expectations up, right I’ll try this [drug]. There’s not anything over the years that they’ve given me that I've refused’. — PAT2C (female, RA) ‘I often don’t really have to say very much because [HCPs are] so clever in, erm, reading people, that they more or less know…I hope in the future we can find a cure for it [RA] (hmm) that would be my expectation of [treatment]…obviously in my lifetime, I mean the doctor I was under actually said to me: “Don’t worry, in 10 years time there’ll be an injection or an infusion.” And as far as I was concerned, that was many years down the line, yet in my lifetime that has happened and it’s come to fruition because, you know, I am a lot better than I was in the very early days when I wasn’t having any infusion at all’. — PAT3D (female, RA) ‘I expect [rituximab] to be effective for a couple more years by the way [drugs] have gone in the past…it’s such a good drug, I think it might be a bit more than five years…They’re the specialists aren’t they after all, and I have to accept whatever they say or do. I know that they’re doing what’s best for me’. — PAT8D (female, RA)	‘I think when [patients] “fail drugs” after that you can have sometimes an open discussion with them about the possible options and they [patients] actually become very open to actually, erm, going into clinical trials (agreement) and all these kinds of things. And so [this hope/interest] makes me actually fight to get them clinical trials in our centre so we can give them options, erm, and that’s what I did recently for Baricitinib because I have a group of patients who “failed” all the biologics that NHS can offer and they just, and they’re begging, they’re ready to go in any clinical trial to try anything which could make their life better’. — HCP5E (male, consultant rheumatologist) ‘Cos we [HCPs have] got the people who are really kind of desperate for their next drug, wanting the next idea’. — HCP4B (female, rheumatology registrar)
HCPs struggle with not being able to ‘fix’ or solve problems for patients, especially PPES		‘In most consultations you will find that something needs some attention, it’s not that you have nothing to offer and everything is already sorted for them [patients]. So either [treatment/advice] will be you can help their pain in some way or some change in the medication to help the inflammation or an injection or, there is usually something that you can improve’. — HCP5D (female, rheumatology registrar) ‘I do think it’s our tendency as health professionals to fix people, to kind of offer a drug as quickly as possible and it’s why [rheumatologists] switch medication rather than exploring other options or engaging the patient. Sometimes the work that needs to be done, should be done by the patient rather than by us [HCPs] if there are other causes for the refractory symptoms’. — HCP1D (female, consultant rheumatologist) ‘Where the work is really rewarding for me is how far you can get with psychological therapy, and that even with the refractory disease, you know where someone has massive struggles physically, they can make such big steps to change their lives’. — HCP6B (female, psychologist)

The difference in presentation/experience resulted in variances in treatment goals, where rheumatologists focused on reducing inflammation or assessing mainly hands, while patients wanted a wider mind–body assessment and to maintain physical function. HCPs are guided by certain thresholds that need to be met to instigate treatment change, which may not reflect those agreed with by patients. These differences in treatment goals were usually based on patients’ illness and treatment beliefs and long-term experiences. However, both patients and HCPs ultimately want to improve quality of life and are working towards the same agreed upon goal, at times achieved by different means.

A related issue was managing patient expectations and realistically explaining the effectiveness of medications and immunological targets, with some symptoms continuing. This balance between optimism and realism can be a difficult but necessary conversation, with patients appreciating honesty. Discordances in goals and expectations may lead to the loss of trust or hope in the prescribed medicines. This loss could result in patients disengaging from services or non-adherence, while HCPs lose optimism for acceptable outcomes for patients due to limited treatment options. Following these negative experiences, difficulties in communication were apparent.

Patients found it difficult to talk about their symptoms/problems, in part due to a lack of time in appointments. HCPs also conveyed the difficulty in understanding patients’ depictions, with faith in their rheumatology team being integral to these clinical interactions. These communication difficulties were not always present, as some patients did mention they could talk openly, ask questions and express their emotions, with enough time given during consultations. However, according to HCPs, the differences in the language used during consultations can lead to misunderstanding and frustration, with patients feeling that they are not being taken seriously by HCPs, as the nuances of terms are not recognized.

Conversely, patients expressed hopeful expectations despite poor experiences, compared with HCPs who reported mainly feelings of hopelessness. Some patients had good relationships with their rheumatology teams built over many years. They trusted the rheumatology staff to provide the level of care expected and had faith that their rheumatologist would find the right treatment. This situation was mirrored by HCPs who had established trustful bonds, due to regular clinic appointments, with those diagnosed with RD. The close rapport allowed patients to develop hope for the next drug to control their disease and symptoms, placing confidence in research and drug development.

Rheumatologists indicated they struggled most with not being able to ‘fix’ or solve problems for patients, by not being able to suppress inflammation through the medications they prescribe and achieve remission, feeling they delivered a subpar service. Several rheumatologists expressed discordant views within this theme that often medication management is not required for some patients. Instead, supporting patients to self-manage their condition is more appropriate through offering helpful evidence-based alternatives or referrals.

### Current problems within managing RD/PPES

The fourth and final theme reflected four current problems that mainly HCPs faced managing this group of patients: lack of clear guidelines/definitions/terms or commissioning/service barriers; lack of evidence on which to base management; RD is uncommon, with only one subtheme that fit with patients’ views as well regarding; and the unpredictable/invisible nature of arthritis/symptoms (see Table 5). Problems with the Polido-Pereira *et al*. definition [30] of RD were highlighted as too vague, with low disease activity not considered an adequate goal. Additionally, this definition implies a ‘treat-to-target’ strategy, therefore not capturing the full problem given that RD/PPES is not limited to the joints of the DAS28.

**Table 5.** Illustrative accounts for theme 4: current problems within managing RD/PPES

Subthemes	HCPs’ accounts	
Lack of clear guidelines or definition, and/or commissioning or service barriers	‘Well, I think that [current definition]’s very vague, isn’t it because low, you know, low disease activity, you know, will take into account VAS scores of some description, be that pain VAS, be that a global wellbeing, be that functional ability and those aren’t purely determined by inflammatory joint disease’. — HCP1E (female, consultant rheumatologist) ‘If you’re using DAS28 then you can get a, you can get a low disease activity score quite happily, cos you're missing out one part, you’re missing out two, it’s a pair of feet (hmm) so I think that’s (pause). From my perspective it’s skewed (hmm and it’s not fully capturing). No, [DAS28 is] not fully capturing the full, the full problem’. HCP1C (female, podiatrist) ‘There are patients who are really refractory to everything and they have dreadful arthritis…I suppose I have the advantage, erm, of working across paediatrics and adults, erm, and actually as soon as they get to 16 I can call them seronegative rheumatoid and then suddenly I’ve got a whole other load of drugs’. — HCP8E (female, consultant rheumatologist) ‘In the adult they rely very heavily on those [DAS28] scores so you cannot even apply for biologics without a certain score, whereas in adolescents, we don’t have to have a certain score in order to be able to access medication’. — HCP2D (female, clinical nurse specialist) ‘We have very rapid, easy access to drug treatment but quite a lengthy wait for psychological support treatments, physiotherapy, occupational therapy is somewhere in the middle probably’. — HCP7E (female, consultant rheumatologist)	
Lack of evidence to base management on	I find myself in this situation where I'm really embarrassed because I don’t know why this drug and not the other one. It’s just because it’s the NHS pathway and, erm, I think patients deserve like better options especially when they get older and they really ask very good questions’. — HCP5E (male, consultant rheumatologist) ‘We [HCPs] don’t have any science comparing drugs, we’ve got none whatsoever. We really haven’t got anything to say whether one biologic’s better than another’. — HCP2E (female, consultant rheumatologist) ‘The specific literature around RA is relatively limited, so, I mean as with a number of other things in clinical health psychology we [Psychologists] do kind of extrapolate a bit. I mean there’s obviously a, a big literature on chronic pain, much less around um, rheumatological issues specifically. Um, so we are needing to extrapolate, um, to some degree, but we are on, kind of, quite solid ground in terms of chronic pain’. — HCP6B (female, psychologist)	
RD not common	‘If we talk about refractory disease that means I have not controlled their inflammation, yes? And actually that’s pretty rare—that’s probably less than 2% of our patients, you know, who have truly refractory disease as I would understand it [the concept of RD], i.e. ongoing inflammation despite biologics, because we have so many biologics available to us [rheumatologists], I think we’re intervening earlier’. — HCP4D (Male, consultant rheumatologist) ‘At least sort of 5 to 10 a month on average with these type of patients that aren’t responding to the current treatment and needing help to manage through erm flare-ups, and then probably a similar sort of number as well who are reasonably well controlled with biologics and DMARDs and things [other drugs], but are needing sort of that chronic pain management approach’. — HCP5C (male, physiotherapist)	

Subthemes	Patients’ accounts	HCPs’ accounts
Unpredictable or invisible nature of RA/symptoms	‘It is unpredictable, but a lot of the time now, there is some kind of thing [symptoms] I’ll know, like I do have some kind of pre-warning signs, let’s say…so yeah, it’s less likely now that they kind of catch me off guard cos I do know kind of what, how it works’. — PAT11D (female, pJIA) ‘I’ve had people say: “Oh my god, I never knew you had arthritis.” To look at me they [people] would think: “Nah, there’s nothing wrong with ya.” Unless they looked at my hands but no-one looks at your hands…but I’ve had a lot of people in the last year mention: “What’s the matter with your neck?” Yeah, and the way I walk, yeah so emotionally that’s got me down’. — PAT4A (female, pJIA)	‘I mean obviously you can't see pain and this is a problem that [HCPs] frequently hear from our patients is that they look well, they’re often young, healthy people, they’re not walking with a stick or a frame, so from the outside it’s not obvious that they’re in high levels of pain’. — HCP2B (male, consultant rheumatologists) ‘Some people do well in pregnancy, other people don’t do well in pregnancy and still they’ll have a lot of the symptoms of arthritis’. — HCP8C (female, occupational therapist)

Paediatric professionals noted differences in treating pJIA compared with RA, including treatment guidelines and reduced medication availability. Although the lack of clear guidelines for pJIA can be frustrating for rheumatologists, there is a level of flexibility compared with the strict RA guidelines. Barriers such as commissioning or services available were discussed, particularly in adult services, where resources are more limited with longer referral lists. Most HCPs reported that another barrier was a lack of evidence to base their management on for RD in RA/pJIA. There is not a clear agreed upon algorithm for which DMARD is better to try next or how to best manage PPES without extrapolating from other conditions.

Therefore, HCPs were unable to deliver evidence-based care and resorted to a trial-and-error approach. Most reflected on the need for more research in this area. Some rheumatologists stated there were enough randomized controlled trials demonstrating DMARD efficacy generally, in contrast to AHPs who felt there were insufficient high-quality trials of non-pharmacological treatments. Many HCPs described RD as uncommon, given the numerous treatment options available, with estimates mentioned in the interviews/focus groups ranging from 1–33%. Hence the rarity of RD contributes to the lack of understanding of underlying mechanisms and appropriate management. However, some HCPs did imply that the number of patients not responding to treatment was common, with people continuing to flare despite changing medication.

A final prominent issue was the unpredictable/invisible nature of arthritis/symptoms, where people did not know which symptoms or joints would be more problematic day-to-day. Patients stated this unpredictability hindered planning and caused misunderstanding about their condition from others. Unpredictability during pregnancy was highlighted specifically, where it is unknown whether RD/PPES will remit or relapse and may require treatment changes. Patients described that living with an invisible/hidden condition or symptoms was hard, while at the same time, having visible aspects such as nodules was difficult.

## Discussion

The aim of this study was to explore patients’ and clinicians’ experiences of treatment in RD/PPES. The objectives were to understand managing and living with RD, including determining expectations and experiences of treatment and the impact on the patient–professional relationship. Four key themes in the management of RD/PPES were identified by both patients and professionals: risk/perpetuating factors/triggers; need for a patient-centred holistic approach to care, diagnosis and treatment; discordance and impact on the patient–practitioner relationship and current problems in managing RD/PPES. These themes covered 22 subthemes, of which 7 were HCP-specific. Risks and triggers of RD/PPES identified by participants covered baseline clinical, biological and lifestyle factors, with RA/pJIA development determined by a complex combination of genetics, hormones and environmental triggers as highlighted by several authors [31, 32].

The most common theme identified was how patients with RD/PPES expected a patient-centred holistic care approach. The MDT and patient support services are important to provide complementary holistic advice on aspects such as quality of life, work/benefits and sex/relationships that patients may not feel comfortable discussing with their rheumatologists [33]. Despite the need to assess/treat psychosocial factors and non-pharmacological treatment options, only a minority of patients are routinely asked about social/emotional difficulties by a rheumatology HCP, but nearly half of patients would welcome discussing its psychological impact [34].

The third theme focused on the discordances between patients and practitioners regarding communication, treatment and expectations, leading to tensions in the patient–practitioner relationship. It is well established that there are often disparities between patient and physician global assessments [7, 17]. Patients tended not to share their problems/symptoms with their clinicians, further supporting the importance of qualitative studies to gain insights that may not be expressed during clinical consultations [35]. Beliefs surrounding treatment expectations link with patients’ illness perceptions and medication beliefs and could be targeted through intervention to respond to patients’ needs [36–38].

The final theme reflected current problems for HCPs in managing these patient groups. Importantly, this study was conducted prior to the EULAR difficult-to-treat initiative, hence why the Polido-Pereira *et al.* RD definition [30] was discussed. There are currently no guidelines for RD in JIA [39], and no clinical recommendations regarding which bDMARD is the most suitable third-line option [1]. Access to MDT services across the UK are not equitable, requiring more specialist nursing and AHP support, especially for paediatric/adolescent rheumatic diseases [40]. Outcome measures do not capture the unpredictability or fluctuating nature of arthritis/symptoms [25]. Therefore, it is important that treatment strategies are appraised and modified as disease activity, illness beliefs and behaviours vary [41].

### Strengths and limitations

This study is one of the first to qualitatively explore RD/PPES in RA/pJIA with patients and HCPs and systematically study both its characteristics and management. Themes identified align with and support the literature on management of difficult-to-treat or refractory RA [42, 43], highlighting their broader application to RD/PPES. HCPs welcomed the opportunity to share their experiences of managing patients with RD/PPES, and a future initiative could explore a case-based conference on RD/PPES to allow further knowledge sharing. The validity and credibility of the study findings was established by following COREQ guidelines, including a thematic map, using direct data from transcripts and using multiple coders [24, 26, 27]. Most themes identified were common to both RA and pJIA, supporting a transdiagnostic approach [44].

There are several limitations to note. Patients and HCPs from England participated, with most recruited from London, therefore findings may not be representative of other UK nations. The inclusion of other relevant HCPs, such as orthopaedic surgeons or optometrists who offer their expertise for joint replacement and extra-articular manifestations like uveitis, would have been useful [45]. However, the ratio of consultant rheumatologists, registrars and specialist nurses to AHPs interviewed reflects the average UK rheumatology department [40]. Additionally, paediatric pharmacists, psychologists, physiotherapists and occupational therapists associated with recruitment sites were approached to take part in the study but did not respond, so targeting these paediatric AHPs through a different recruitment strategy may be required in the future.

Age at diagnosis in this sample may be considered below average for RA [interquartile range (IQR) 24–48.8 years], potentially influencing the derived themes. However, a worldwide analysis of 44 countries (*n* = 2481) identified that in 28% of patients RA began before age 36 years and in 50% before age 46 years [46], indicating that the patient sample may be representative of those diagnosed at a younger age. It may be prudent to conduct future research in those who are diagnosed later in life to explore any further challenges regarding RD/PPES.

Having DAS/JADAS in the inclusion criteria was a barrier to recruiting those with PPES and pJIA, as DAS/JADAS scores were not routinely determined since they are not required for treatment escalation. The National Early Inflammatory Arthritis Audit analysis showed that 34.3% of DAS28 data were missing at critical management time points [47], with missing data from younger patients, not RF positive and with longer symptom duration, therefore most likely to be pJIA patients. The authors of that study suggest the missing data were due to a focus on collecting data for ‘typical’ inflammatory or severe symptoms [47], hence why those with PPES did not have DAS28 scores recorded.

## Conclusion

It is clear from this qualitative exploration that future research and potential novel management strategies need to be developed to provide an appropriate treatment plan based on specific experiences of RD/PPES. A patient-centred holistic approach to care, diagnosis and treatment is important when managing patients with RD/PPES, and patient–HCP discordance can occur when management expectations do not align. Specialist RD/PPES clinics and case-based conferences could be a starting point to discuss and agree upon appropriate management in conjunction with patients and enable sharing of best practices.

## Supplementary Material

rkae076_Supplementary_Data

## Data Availability

The majority of the qualitative data is contained within Supplementary Data S2, available at *Rheumatology Advances in Practice* online (Framework) and further data are available upon request from the corresponding author.
