# Supplementary material for: Management of refractory disease and persistent symptoms in inflammatory arthritis: qualitative framework analysis of interviews with patients and healthcare professionals
Source: Rheumatol Adv Pract. 2024 Jun 10;8(3):rkae076. doi: 10.1093/rap/rkae076 (PMC11223812; doi:10.1093/rap/rkae076)
Supplement: rkae076_Supplementary_Data [file rkae076_supplementary_data.zip › 23-218 Supplementary Figure S1.docx]

### Supplementary Figure S1: Overview of Framework for Management of RD/PPES.

A coloured cell indicates it contains an account from that participant for that category.
