# Supplementary material for: Management of refractory disease and persistent symptoms in inflammatory arthritis: qualitative framework analysis of interviews with patients and healthcare professionals
Source: Rheumatol Adv Pract. 2024 Jun 10;8(3):rkae076. doi: 10.1093/rap/rkae076 (PMC11223812; doi:10.1093/rap/rkae076)
Supplement: rkae076_Supplementary_Data [file rkae076_supplementary_data.zip › 23-218 Supplementary Table S1.docx]

**Supplementary Table S1**: Inclusion and Exclusion Criteria

| Patients | |
| --- | --- |
| Inclusion Criteria | Exclusion Criteria |
| 1. Diagnosis of Inflammatory Arthritis, e.g. Polyarticular JIA or RA 2. Established disease with duration longer than 2 years 3. Aged 16 years old and above 4. Patients under the care of a rheumatologist at recruiting sites attending outpatient rheumatology clinics 5. RD: Moderately active disease as defined by either Disease Activity Score-28 Joints (DAS28) >3.2, JADAS10 >3.8 or PPES: if Patient Global Assessment score ≥5/10 and DAS28 >2.6 or JADAS10>1.0 in the last 3-6 months 6. Previously didn’t respond to two Disease Modifying Anti-Rheumatic Drugs (DMARDs) and currently on one biologic 7. Experiencing persistent physical and emotional symptoms such as pain and fatigue, lasting for at least three months and affecting functioning | 1. Diagnosis of other rheumatic disease (e.g. Other subtypes of Juvenile Idiopathic Arthritis or Osteoarthritis) 2. Early disease with duration less than 2 years 3. Under 16 years of age 4. Severe co-morbidities such as mental health or respiratory issues e.g. schizophrenia, severe depression, pneumonia, 5. Participants unable to give written informed consent, complete questionnaires or participate effectively in the interview due to:   a. Insufficient command of English  b. Significant learning disability  c. Reduced cognitive capacity |
| Healthcare Professionals | |
| Inclusion Criteria | Exclusion Criteria |
| Healthcare professionals (e.g. Consultants, Nurses and Allied Health Professionals) who are currently working in Rheumatology across recruiting sites have worked in Rheumatology for >1 year. | Unqualified healthcare professionals who are currently working in Rheumatology across the study sites, or have not worked in Rheumatology for <1 year. |
