# Supplementary material for: Management of refractory disease and persistent symptoms in inflammatory arthritis: qualitative framework analysis of interviews with patients and healthcare professionals
Source: Rheumatol Adv Pract. 2024 Jun 10;8(3):rkae076. doi: 10.1093/rap/rkae076 (PMC11223812; doi:10.1093/rap/rkae076)

### Supplementary Data S1: A) Patient Interview Schedule


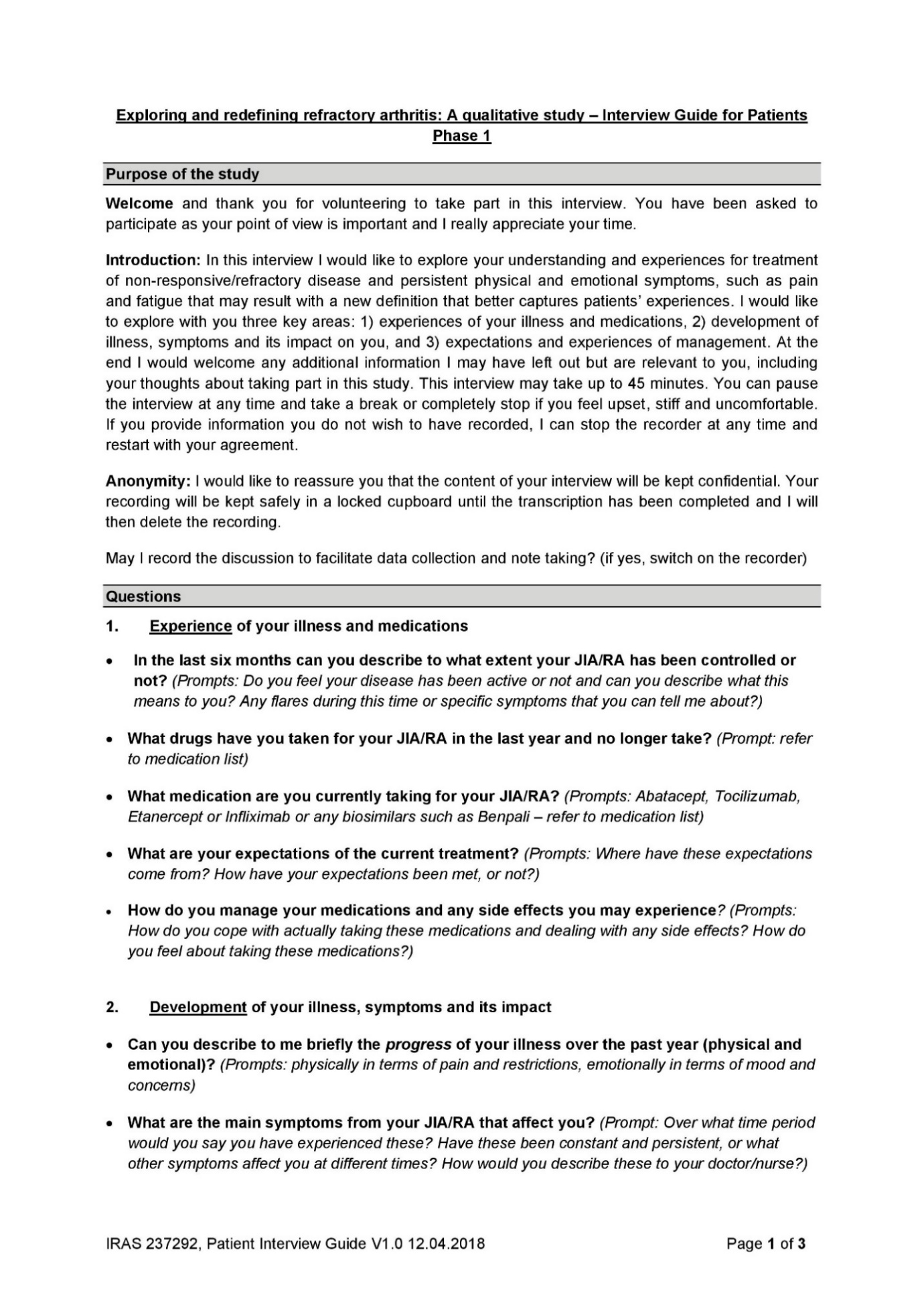


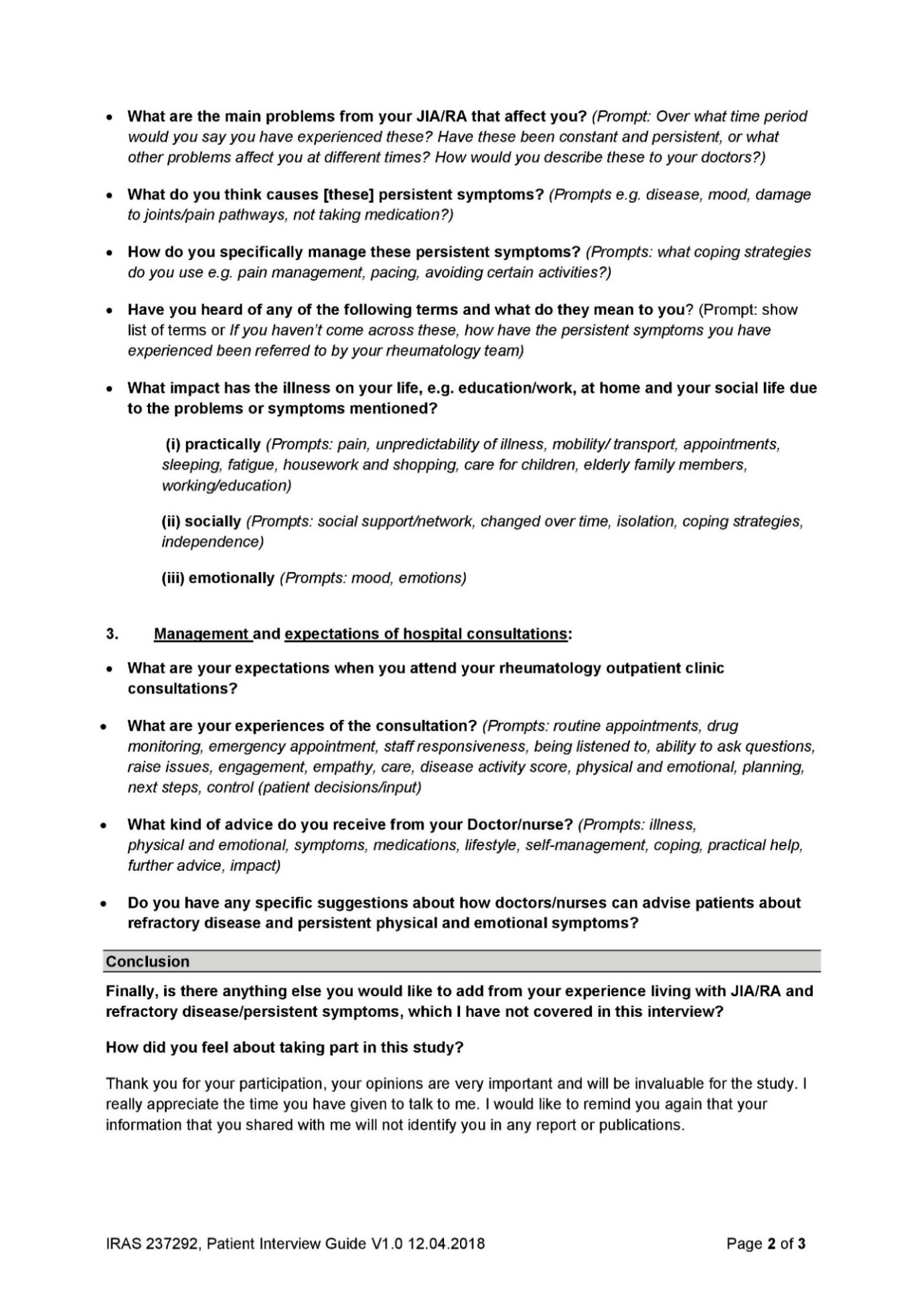


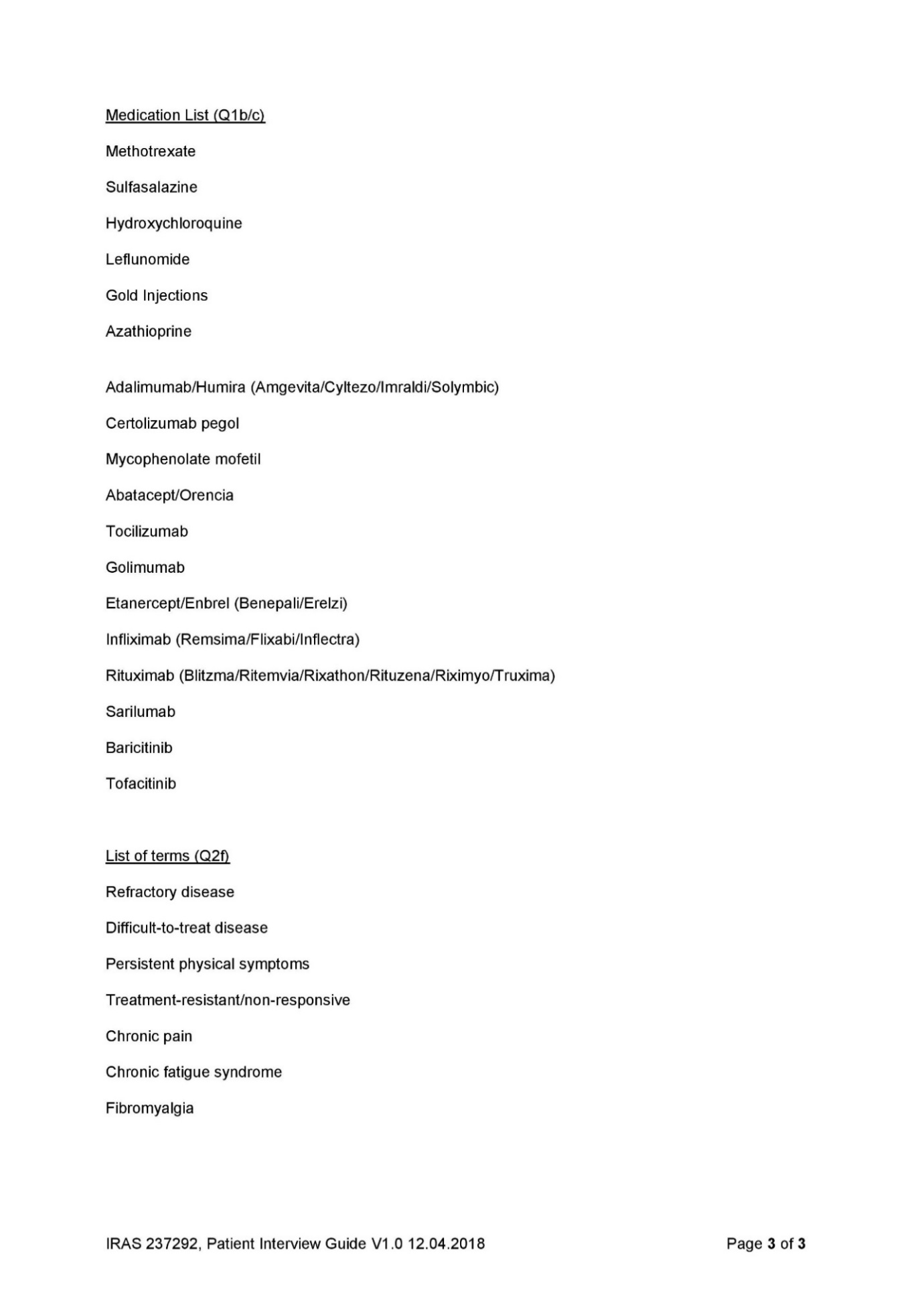


### Supplementary Data S1: B) HCP Interview Schedule


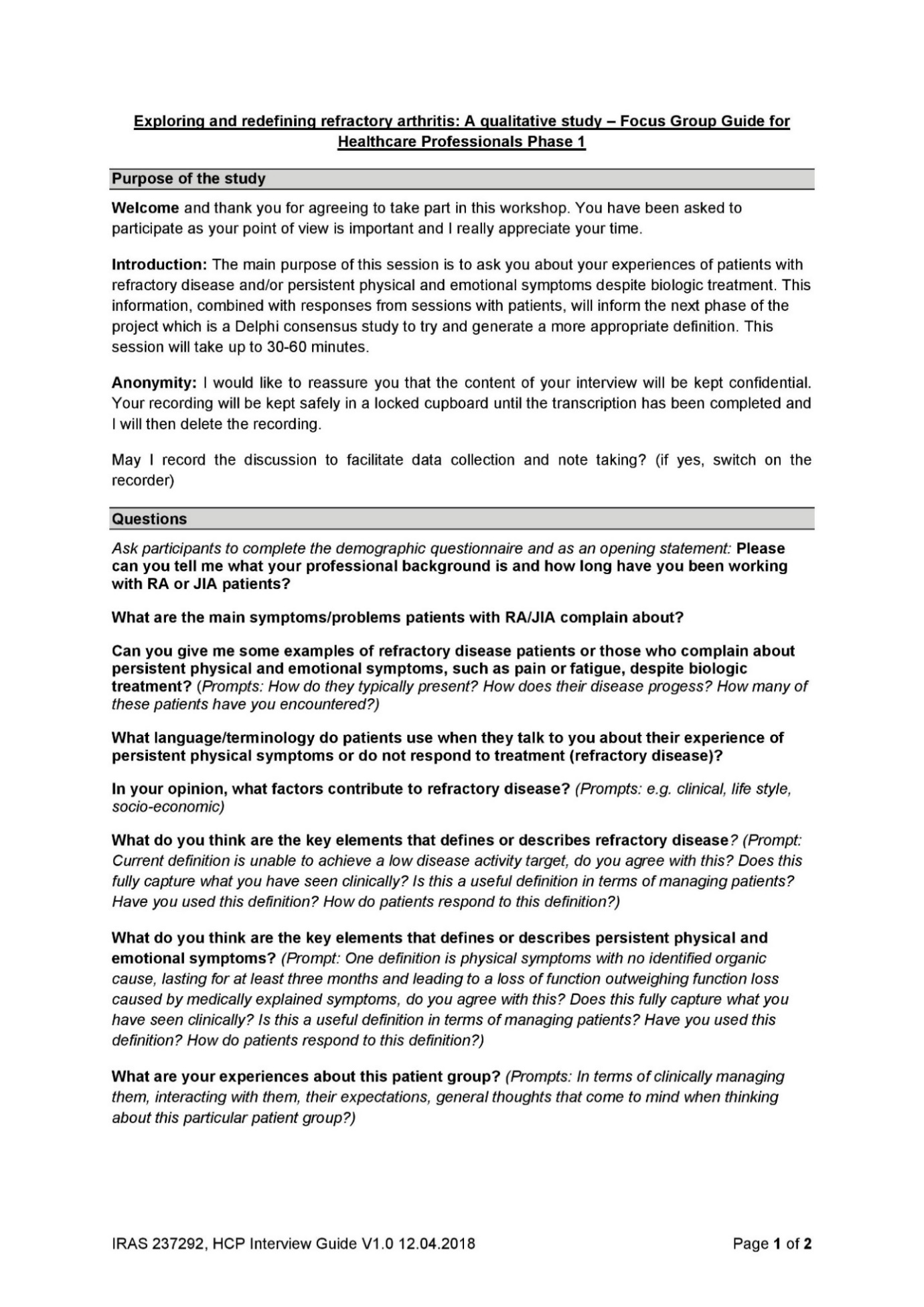


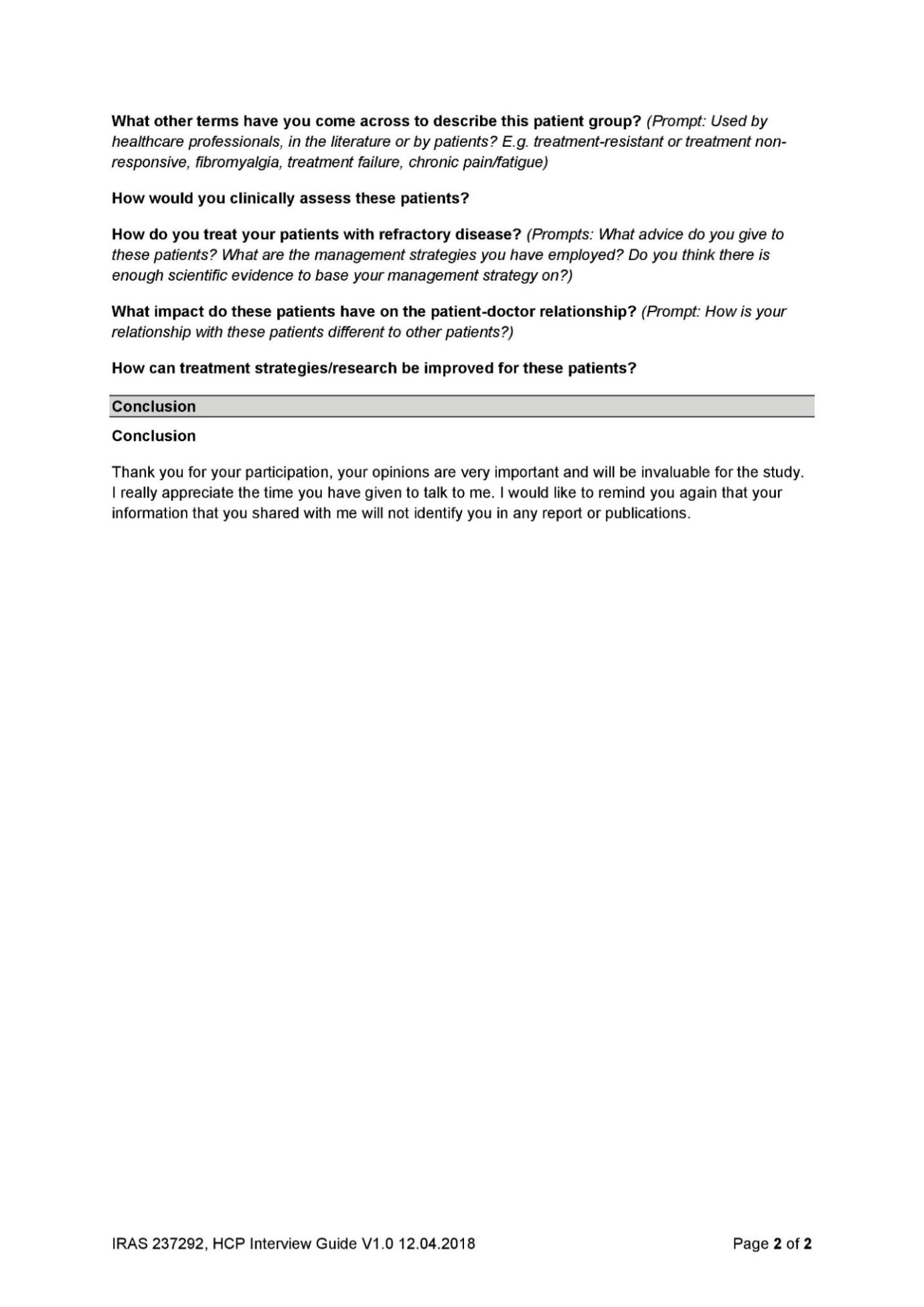

Supplement: rkae076_Supplementary_Data [file rkae076_supplementary_data.zip › 23-218 Supplementary Data S1.docx]
